# Non-Occlusive Mesenteric Ischemia in Cardiac Arrest Patients

**DOI:** 10.31083/j.rcm2409262

**Published:** 2023-09-19

**Authors:** Jana Smalcova, Jan Belohlavek

**Affiliations:** ^1^Anaesthesiology and Resuscitation Department, Cardiac Centre, Institute for Clinical and Experimental Medicine, 14000 Prague, Czech Republic; ^2^First Faculty of Medicine, Charles University, 12108 Prague, Czech Republic; ^3^2nd Department of Medicine – Department of Cardiovascular Medicine, General University Hospital, 12800 Prague, Czech Republic

**Keywords:** non-occlusive mesenteric ischemia, cardiac arrest, ischemia-reperfusion injury

## Abstract

Non-occlusive mesenteric ischemia (NOMI) is a severe complication in patients after cardiac arrest (CA). The diagnosis is complicated, the treatment options are limited. Given the susceptibility of enterocytes to ischemia, the incidence and severity of NOMI in the post-resuscitation period may reflect the intensity and duration of both ischemia and subsequent reperfusion injury. NOMI is considered to be associated with adverse neurological outcomes in CA patients. Therefore, NOMI should not only be regarded as a post-resuscitation complication but also as one of the prognostic markers in CA patients.This paper summarizes current knowledge on NOMI’s pathophysiology, diagnosis, treatment, and prognostic significance in CA patients.

## 1. Introduction

**Fig. 1.**
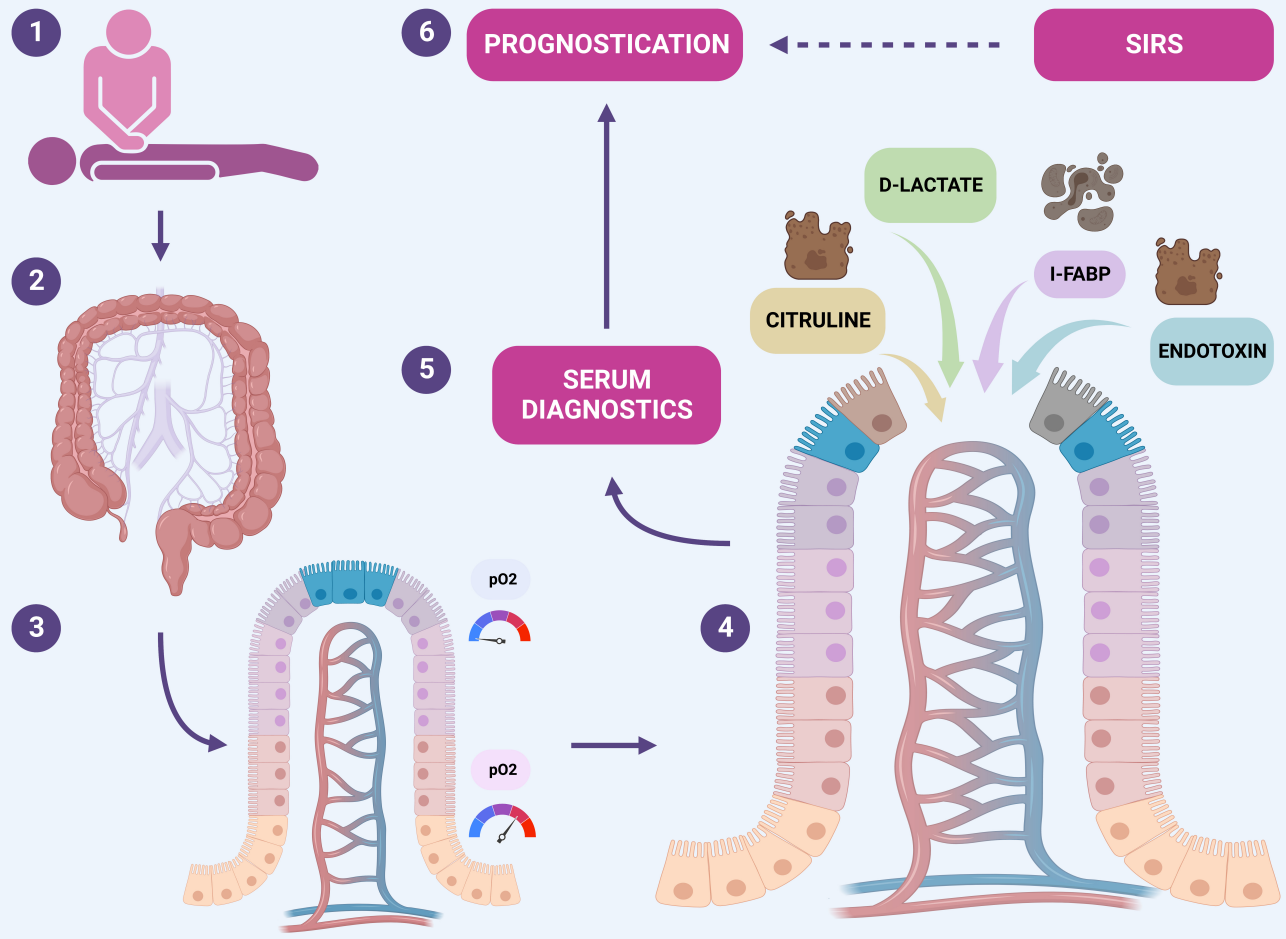
**NOMI and pathophysiology**. (1) Cardiac arrest is a serious condition for which CPR is the solution. (2) During CPR, hypoperfusion of all organs, including the intestine, occurs. (3) Intestinal villi are very vulnerable to IR damage due to the arrangement of the vascular supply to the villi. (4) During IR injury, the endothelial structure of the villi is disrupted, and some substances are absorbed into the bloodstream and detectable in the serum. (5) The diagnosis is based on the detection of these serum markers. (6) The degree of IR damage reflects the severity of the hypoperfusion and hypoxia, the intensity of SIRS, and can be used to assess neurological prognosis. CPR, cardiopulmonary resuscitation; IR, ischemia-reperfusion; SIRS, systemic inflammatory response syndrome; I-FABP, intestinal fatty acid-binding protein; NOMI, non-occlusive mesenteric ischemia; pO2, partial pressure of oxygen. Created with https://www.biorender.com/.

Non-occlusive mesenteric ischemia (NOMI) belongs to the serious and probably underestimated complications in critically ill patients, including cardiac arrest (CA) patients. NOMI has been described in hemodynamically compromised patients with all forms of shock and accounts for 5 to 30% of all acute mesenteric ischemia cases [1, 2]. It is a general reaction of the intestine to severe hypoperfusion with subsequent reperfusion reaction, regardless of the cause. Intestinal damage with all its consequences can be observed in patients with low cardiac output syndrome, cardiogenic shock, hemorrhagic shock, septic shock, in cardiac surgery patients and following cardiac arrest [3]. Regarding the duration of hypoperfusion, patients with prolonged CA represent a subgroup with a high risk of ischemia-reperfusion (IR) damage [4]. The body suffers from IR injury during cardiopulmonary resuscitation (CPR), which is directly related to the time and quality of CPR. NOMI may affect 2.5 to 6% of these CA patients [5]. The mortality rate is usually very high, ranging from 50 to 93% [6, 7, 8]. Non-occlusive intestinal ischemia is characterized by the absence of mechanical obstruction, like an embolic or thrombotic occlusion of the mesenteric arteries [9]. It may result in intestinal cell dysfunction and transmural necrosis of the bowel [1, 10]. The cause of this condition is mostly related to hypoperfusion of the intestine due to low cardiac output, spasm of mesenteric vessels, hypovolemia, or use of vasoconstrictive agents [7, 8], which can significantly reduce the perfusion of the intestine and may result in transmural necrosis [11, 12, 13]. The enterocytes are very sensitive to ischemia-reperfusion injury. In the period of CA, transient hypoperfusion of all organs and deterioration of the microcirculation, which is responsible for oxygen and nutrient exchange, can lead to the worsening of the integrity of the gut barrier and contribute to the possible transmigration of bacteria and endotoxemia [14, 15]. Thus, depending on the duration of CA, especially in patients with prolonged CA, this process can aggravate a systemic inflammatory reaction with increased production of pro-inflammatory cytokines [16, 17]. Regarding the hypoperfusion and reperfusion that occur during cardiac arrest, NOMI is not only a severe complication of a critical state, but its incidence and severity are considered to be prognostic markers of CA outcome [18]. This review summarizes the current information about NOMI in CA and its potential significance and association with the prognosis of post-resuscitation status (Fig. 1).

## 2. Physiology and Pathophysiology of Intestinal Non-Occlusive Ischemia

Currently, the exact pathophysiology of NOMI is still not fully understood. However, it is probably closely associated with the splanchnic blood flow reduction in shock conditions and the use of vasopressors, see the Fig. 1.

### 2.1 Physiology of the Intestine 

The gastrointestinal tract is supplied by three main arteries: the celiac trunk and the superior and inferior mesenteric arteries. These arteries split into the plexuses: serosal, submucosal and mucosal. The splanchnic vascular bed receives 25% of the cardiac output and is responsible for most blood delivery to the mucosa and submucosa. Sixty-five to seventy-five percent of the total intestinal blood flow is distributed to the intestinal mucosa at rest [19], primarily because of high metabolic demands. Approximately 90% of total intestinal blood flow is distributed to the mucosa during maximal vasodilatation [20]. A specific arrangement of arteries and veins exists in the intestinal villi. They flow parallel in the opposite direction, and they are connected through a tight capillary network. This structure causes a decline in oxygen saturation from the villi’s base up to its top, and it could be the leading cause of the intestine’s susceptibility to hypoxic injury.

### 2.2 Intestinal Susceptibility to Ischemia

In order to maintain blood flow to the brain and heart during acute hypotension, systemic autoregulation can overwhelm the protective mechanisms of bowel local autoregulation [21]. The mucosa of the intestinal wall is the layer most susceptible to the effects of ischemia. The splanchnic region participates in the regulation of circulating blood volume and systemic blood pressure. Blood flow to vital organs is maintained by shifting the flow away from the splanchnic vessels. As a consequence, any significant reduction of splanchnic blood flow can be vital in acute hypoperfusion of the heart or brain. Blood flow through the mesenteric artery is proportional to blood pressure. Despite this, previous atherosclerotic involvement of the splanchnic circulation may exacerbate the severity of ischemic involvement [22]. In addition, during the shock condition, the blood supply to the intestinal mucosa is significantly reduced due to sympathetic stimulation. Ischemia damage starts from the mucosa and continues towards the serosa. Subsequent reperfusion of the intestine results in further damage to the mucosa [23]. In case of shock, the renin-angiotensin system promotes a relative increase in mesenteric vascular tone compared to other regional vessels [24]. Mesenteric vasoconstriction is usually observed as early as 10 minutes after the onset of hypotension [25]. Furthermore, lactate production increases due to anaerobic glycolysis in response to decreased oxygen uptake below demands. In case of the vasopressor use, the microcirculatory changes result from the alpha-adrenoreceptor stimulation [26, 27] and can persist even after intestinal blood flow returns to normal [28]. Another drug that can affect the microcirculatory flow is furosemide. After furosemide administration, the increased renal blood flow leads to diminished mesenteric perfusion. This may well happen probably due to the furosemide-related activation of the renin-angiotensin system with increased levels of angiotensin II [25, 29]. However, persistent intestinal hypoperfusion leads to hypoxic bowel injury, which likely contributes to the development of organ failure and an increase in intensive care unit (ICU) patient mortality. Increased permeability of damaged mucosa and capillary endothelium can cause water and macromolecular leak into the intestinal wall, resulting in intestinal wall thickening [30]. After 6 hours from 30 minutes of ischemia, it is possible to observe a statistically significant decline in crypt-villus heights compared to the corresponding non-ischemic reference specimens, and even more after 60 minutes of ischemia. In addition, mucosal alterations associated with ischemia-reperfusion injury are apparent and more expressed in differentiated enterocytes [31]. In the early phase, the ischemic mucosal injury might be reversible. The transmural injury was described after four to six hours of ischemia [32, 33], which induced a secondary systemic inflammatory response syndrome (SIRS).

### 2.3 Intestinal Barrier Damage

The functional intestine barrier is crucial to prevent systemic microbes and toxins contamination. The reliability of the intestinal barrier depends on the proper function of epithelial components. Failure of the barrier function could allow bacteria or microbial products, such as endotoxin or flagellin, to enter the systemic circulation, thereby amplifying the inflammatory response [34, 35]. Ischemia-reperfusion injury predominantly affects the intestinal mucosa and submucosa due to oxidative stress and inflammation, and impairs the mechanisms that prevent the translocation of bacteria from the intestinal lumen [24, 36, 37]. During recovery, the bowel may not be functional, and attempts at enteral feeding may result in intestinal distension, osmotic diarrhea, and additional intestinal damage [33]. Therefore, enteral nutrition should be cautiously administered or delayed in patients with severe heart failure being treated with dobutamine or in cases of multi-organ failure [38]. Similarly, in extracorporeal cardiopulmonary resuscitation (ECPR), delayed enteral nutrition has been associated with improved neurologically favorable survival [39].

## 3. Diagnostics

Identification of NOMI in CA patients remains difficult due to the absence of reliable and specific markers which can indicate intestinal dysfunction.

### 3.1 Clinical Symptoms

The risk of developing NOMI increases with age, the length of CPR and its quality. Surprisingly, atherosclerosis of the mesenteric arteries is not one of the risk factors for the severity of IR intestine injury [40]. The clinical signs of NOMI are not specific enough, as early symptoms are frequently absent. In addition, patients post-CPR are, in most cases, sedated for days after the event [9, 41]. As a result, NOMI is frequently diagnosed in advanced stages. Thus, the first symptom may be profuse diarrhoea or increased waste from the nasogastric tube, which are non-specific signs of intestinal dysfunction. NOMI could be further suspected in patients with the following symptoms mainly gastrointestinal bleeding, abdominal distension and abdominal mottling [5]. In the early stages of the disease, neither the visceral nor the parietal peritoneum is affected [42]. The early onset of profuse diarrhea (<12 h) as a manifestation of ischemia-reperfusion injury has been described in patients with CA and return of spontaneous circulation (ROSC) and was associated with poor neurological outcomes [18, 43]. Similar results were observed in patients with prolonged CA [6, 44]. Although higher lactate and base excess values with lactic acidosis in patients post-CPR are non-specific signs, these values are considered alarming in terms of possible NOMI development [9].

### 3.2 Examination Methods

Unfortunately, imaging methods are only of limited value for early diagnosis of NOMI.

Ultrasound is a readily available, non-invasive technique whose application in the case of NOMI is highly limited. For instance, distended intestinal bowel loops and hypoechogenic bowel wall thickening due to edema or fluid collections may be observed, but none of these findings is specific to NOMI [2].

Computed tomography (CT) can be used to detect intestinal ischemia, but several factors may also limit the value of this radiological examination. CT is helpful in the recognition of transmural intestinal necrosis and in excluding arterial occlusion [45, 46]. Unfortunately, the signs of non-occlusive intestinal ischemia are scarce in the early phase [47, 48]. Non-occlusive ischaemia of the large intestine typically manifests as ischaemic colitis. CT scan may show mural thickening, pericolic fat stranding, and mucosal hyperenhancement [46, 49]. Small bowel ischemia usually presents as a lack of wall enhancement and dilation. Identifying intestinal necrosis is crucial for further treatment, whether a surgical or conservative approach is chosen [46]. On the other hand, several further factors may limit the value of radiological examinations of CA patients. First, some patients are hemodynamically unstable initially, therefore, transportation for a CT scan can be rather complicated or even impossible. More than 40% of CA patients suffer from acute kidney injury due to IR damage [50, 51]. Furthermore, the majority of them undergo early coronary angiography because cardiac causes of CA are among the most frequent [44, 52]. Consequently, the concurrent administration of contrast media could precipitate acute kidney injury. The same problem can be observed with mesenteric angiography, the method of choice in diagnosis of acute mesenteric ischemia. Endoscopic methods for the upper or lower gastrointestinal tract may reveal mucosal lesions caused by IR injury [52]. Therefore, we can expect these lesions, especially in patients with prolonged CA, with higher doses of epinephrine or asystole. Despite its accessibility, the endoscopic examination also has several limitations. One of these is the unavailability of the small intestine. Moreover, mucosal necrosis and transmural necrosis do not always correspond. Inadequate preparation of the bowels can also complicate the examination, and the risk of perforation cannot be excluded [53].

## 4. Biomarkers

Laboratory tests still mostly rely on conventional, non-specific systemic biological markers like lactate and acid-base status. None of these biochemical tests is specific for NOMI.

The higher frequency of abnormalities in biomarkers could support the suspicion of gastrointestinal tract damage after CA. The most promising biomarkers associated with mucosal ischemia are intestinal fatty acid binding protein (I-FABP), D-lactate or citrulline [54]. The routine use of these promising biomarkers is still clinically limited. The most specific biomarkers rise when mesenteric ischemia develops to a late stage [55]. At present, not much is known about the usage of these markers in CA patients. They have primarily been studied in smaller patient groups, and additional research results are expected [56].

### 4.1 Fatty Acid-Binding Protein

This group of proteins is being investigated as a potential biomarker in various medical disciplines. The specific intestinal isoform (intestinal fatty acid-binding protein (I-FABP)) is a cytosolic protein expressed in mature enterocytes located at the tips of the intestinal villi, the areas most vulnerable to ischemia. The normal serum I-FABP levels span 8.33 ± 6.25 ng/mL [57]. In ischemic damage, this protein is quickly released into circulation. As a result, its serum levels increase from shallow standard to easily measurable levels. These may then reflect the severity of ischemia-reperfusion injury [54]. Several studies indicate that the sensitivity and specificity for mesenteric ischemia diagnosis are approximately (80–90% and 85–89%), respectively [15, 57]. In CA patients, FABP may also be used as a prognostic indicator. Some studies show that a higher baseline I-FABP value is associated with adverse neurological outcomes and prognosis [18, 58, 59]. A single-centre study of 69 patients admitted for CA showed that I-FABP levels were very high at admission but nearly undetectable the following day [18]. In one of the recent studies, the association between I-FABP, multiple organ dysfunction, and 30-day mortality was observed. In a cohort of 50 patients, elevated admission I-FABP levels (38 ng/L) were associated with a higher incidence of multiple organ dysfunction and mortality. Conversely, the mean I-FABP values at admission in patients with a better prognosis were 18.3 ng/L [56]. I-FABP can be detected by ELISA in serum or urine. However, these tests are not usually available in routine practice. FABP isoforms, different from intestinal FABP, are released from the brain tissue in cases of cerebral ischemia. The extent to which the values of these markers correspond to those of the intestine and the extent to which these values may support the prognostic significance of FABP are the subjects of ongoing research [60, 61, 62].

### 4.2 Citrulline 

Plasma citrulline is a non-protein amino acid. It is predominantly produced by enterocytes of the small intestinal mucosa. It is known as a functional enterocyte mass marker. The average plasma concentration is about 40 umol/L [63, 64]. Most CA patients suffer from ischemia-reperfusion injury of the small intestine. Therefore, during the first 24 hours after CA, it is possible to observe decreased citrulline levels in these patients. Low plasma citrulline is mainly associated with elevated I-FABP concentrations and bacterial translocation [59]. This effect is likely to be more pronounced in patients with prolonged CA due to the severity of ischaemia and subsequent reperfusion injury. However, further research is also needed.

### 4.3 D-Lactate 

D-lactate may be an indicator of splanchnic hypoperfusion [65]. It is an isomeric form of lactate produced by colic bacteria as a typical result of bacterial metabolism. The normal level of D-lactate is around 5.47 ± 1.64 ug/mL. However, during ischemia, as the usual mucosal barrier is injured and permeability rises, D-lactate is released into the circulation. Since the liver cannot metabolize D-lactate due to a lack of D-lactate dehydrogenase, a higher blood concentration can be detected [57, 66]. It may also reflect the intensity of bacterial translocation [67].

### 4.4 Endotoxin

Endotoxin (lipopolysaccharide or LPS) is a major component of Gram-negative bacterial membranes and is common in the human intestine [59, 67]. The average plasma concentration is approximately 3 pg/mL. If released into the circulation, it causes multiple toxic effects, primarily by activating toll-like receptor 4 (TLR4). The most significant reactions are leukocyte and immune system activation to produce pro-inflammatory cytokines and activation of the complement and coagulation systems. Endotoxemia, sepsis, or the exacerbation of the systemic inflammatory response result from the release of a large amount of endotoxin [68]. In the case of intestinal barrier injury, increased motility of the gastrointestinal tract as a consequence of nutritional administration may increase endotoxin translocation.

In association with endotoxemia, higher levels of biomarkers, such as I-FABP or citrulline, have been observed in patients after CA [59].

## 5. Prognostic Implications

In CA patients, the incidence of NOMI and some specific biomarkers have been studied in relation to their prognosis. Non-occlusive mesenteric ischemia can occur in up to 2.5–6% of patients following CA and is usually associated with high mortality [5]. Clinically, among other signs, like gastrointestinal hemorrhage, vomiting or abdominal distension, NOMI can manifest with early diarrhoea (<12 h). Diarrhoea can be considered an early sign of significant IR damage. Adverse neurological outcomes were observed in almost 70% of CA patients with ROSC who presented with clinical signs of NOMI [44].

In addition, studies suggest a connection between ischemic lesions of the upper gastrointestinal tract and adverse neurologic outcomes [69]. High lactate, low pH and base excess, and a high catecholamine dose are often cited as negative prognostic factors [70, 71]. By multivariate analysis, cardiovascular comorbidities, female sex, initial lactate >5 mmol/L, low flow >17 minutes, and inotropic score >7 ug/kg/min were significantly associated with a high risk of NOMI [5]. Some studies also emphasize the prognostic significance of biomarkers like I-FABP, D-lactate, and citrulline [57, 72, 73]. These markers may indicate the severity of ischemia-reperfusion injury to the intestine, which is highly sensitive to hypoperfusion and hypoxia. The course of post-resuscitation illness and the patient’s prognosis may be subsequently affected by damage to the IR and intestinal barrier.

## 6. Treatment

The most important moment, in terms of treatment, is the early and correct diagnosis. Determining whether the patient has non-occlusive ischaemia or acute vascular occlusion is crucial to the patient’s prognosis, as is the diagnosis of evolving intestinal necrosis. Early recognition and correction of vascular pathology are the only ways to reduce NOMI’s high morbidity and mortality [2]. The initial goal of the treatment is hemodynamic stability and the minimal dosing of systemic vasoconstrictors [1]. Surgical intervention may be necessary if the ischemic damage progresses. There are several studies on the surgical treatment of NOMI. In the early stages of NOMI, surgical intervention is not recommended when bowel ischemia is incomplete [13, 33, 74]. In more advanced stages after the onset of intestinal necrosis, patients with surgical resection of the bowel had a better prognosis, but this depended on the length of the resected bowel [75]. The Sequential Organ Failure Assessment score (SOFA score) and some biological markers such as lactate, total bilirubin, lactate dehydrogenase, or albumin may be possible predictors to avoid unnecessary laparotomy and to choose conservative therapy [76].

Vasodilators are currently being studied to affect vasospasm and vasoconstriction of the mesenteric vessels. In addition, a reduction of lactate levels associated with improved survival has been described in patients treated with continuous intravenous prostaglandin [71, 76] or local intra-arterial papaverine administration [70]. Although evolving principles and treatment options exist, additional research will be required to establish an effective and reliable therapy.

## 7. Conclusions

Non-occlusive intestinal ischaemia in CA patients is likely to be a lesser-known complication with an incompletely understood pathophysiology. Nevertheless, it may severely impair the post-resuscitation course. Despite this clinically critical aspect in the post-resuscitation period, the significance of NOMI seems to be more important in the prognostication of CA patients. The incidence and course of NOMI reflect the severity of IR injury in the peri-resuscitation period. Moreover, we can quantify this damage using biomarkers, which may help improve the neuroprognostication of CA patients, especially those with prolonged CA. Yet, from this point of view, more studies and observations are needed.
